# Antimicrobial Resistance and Migration: Interrelation Between Two Hot Topics in Global Health

**DOI:** 10.5334/aogh.4628

**Published:** 2025-03-06

**Authors:** Sergio Cotugno, Elda De Vita, Luisa Frallonardo, Roberta Novara, Roberta Papagni, Muhammad Asaduzzaman, Francesco Vladimiro Segala, Nicola Veronese, Emanuele Nicastri, Anna Morea, Ferenc Balázs Farkas, Botond Lakatos, Roberta Iatta, Giovanni Putoto, Annalisa Saracino, Francesco Di Gennaro

**Affiliations:** 1 Department of Precision and Regenerative Medicine and Ionian Area (DiMePRe‑J), Unit of Infectious Diseases, University of Bari ‘A. Moro’, Polyclinic Hospital, Bari, Italy; 2 Department of Community Medicine and Global Health, Institute of Health and Society, Faculty of Medicine, University of Oslo, Oslo, Norway; 3 Saint Camillus International University of Health Sciences, Rome, Italy; 4 Clinical and Research Infectious Diseases Department, National Institute for Infectious Diseases, Lazzaro Spallanzani IRCCS, 00149, Rome, Italy; 5 Interdisciplinary Department of Medicine, University of Bari, Bari, Italy; 6 Institute of Medical Microbiology, Faculty of Medicine, Semmelweis University, Budapest, Hungary; Pediatric Center, Semmelweis University, Budapest, Hungary; 7 Semmelweis University Department of Internal Medicine and Hematology, Departmental Group of Infectious Diseases, Budapest, Hungary; 8 Operational Research Unit, Doctors with Africa CUAMM, Padova, Italy

**Keywords:** migrants, antimicrobial resistance, global health, One Health perspective

## Abstract

*Background:* Antimicrobial resistance (AMR) and migration are two interlinked issues and both pose an escalating threat to global health. With an increasing trend, there are 281 million migrants globally, while AMR is contributing to over 5 million deaths annually, with a projected rise to 10 million by 2050 if left unaddressed. Both AMR and migration are multifaceted problems that extend beyond human health, involving animals, plants, and the environment—a fact highlighted by the One Health approach.

*Objective:* The aim of this work is: (1) to examine the complex relationship between migration and AMR, drawing on epidemiological data, surveillance strategies, and healthcare access challenges and (2) to address an interventional strategy proposal.

*Methods:* We performed a narrative review of the most updated literature about migration and AMR using three primary databases: PubMed, Scopus, and Embase.

*Findings:* Migrants, particularly from low‑ and middle‑income countries, represent a unique group at increased risk of AMR due to factors such as overcrowded living conditions, limited access to healthcare, uncontrolled use of antibiotics, and high prevalence of AMR in origin countries. Studies reveal higher rates of AMR colonization and infection among migrants compared with native populations, with specific pathogens such as MRSA and multidrug‑resistant gram‑negative bacteria posing significant risks. Migratory conditions, socioeconomic vulnerability, and healthcare barriers contribute to this heightened risk.

*Conclusion:* To address the intersection of migration and AMR, interventions must focus on improving living conditions, enhancing healthcare access, promoting appropriate antibiotic use, and strengthening microbiological surveillance. Multisectoral collaboration is essential to mitigate the spread of AMR and safeguard both migrant and global public health.

## 1. Introduction

Antimicrobial resistance (AMR) is a major global health threat that requires multidisciplinary and complex understanding. Given its profound implications, the issue has been accorded the highest priority on the agenda of the World Health Organization (WHO). It is estimated that drug‑resistant infections contribute to more than 5 million deaths annually. Without timely and effective interventions to curb the proliferation of AMR, particularly in low‑ and middle‑income settings, this grim figure could potentially double to 10 million deaths per year by 2050 [1]. AMR is defined as the ability of bacteria, viruses, fungi, and parasites to resist the effects of antimicrobial agents, making infections harder to treat and increasing the risk of disease spread, severe illness, mortality (particularly in children and the elderly), and healthcare costs [2].

AMR is a complex phenomenon. Both genotypic and phenotypic analyses are used to profile pathogens and pathogen‑oriented infection control measures. Through these evaluations, some categories are established to help in understanding AMR. The WHO Bacterial Pathogens List was first issued in 2017 to strengthen the global antibacterial surveillance and targeted interventions. Recent updates stress the importance of multiresistant gram‑negative bacteria (carbapenem‑resistant *Acinetobacter baumannii*, third generation resistant cephalosporin and carbapenem‑resistant Enterobacterales) and rifampicin‑resistant *Mycobacterium tuberculosis* listing as of critical priority. This has been based on the high resistance‑transfer potential, the severity of infections from clinical point of view and high burden of public health consequences. Pathogens with high priority were identified as fluoroquinolone‑resistant *Salmonella Typhi*, nontyphoidal *Salmonella spp, Shigella spp, Neisseria gonorrhoeae*, vancomycin‑resistant *Enterococcus faecium*, carbapenem‑resistant *Pseudomonas aeruginosa*, and methicillin‑resistant Staphylococcus aureus [3]. This categorization and these definitions allow for epidemiological reporting and empirical treatment strategies. Another key consideration in addressing AMR is that resistance occurs not only in infected patients but also in colonized subjects. Colonization refers to the presence of a specific microorganism within an individual’s microbiome without causing symptoms [2, 4]. When the microbiome includes drug‑resistant bacteria, the asymptomatic patient can unknowingly contribute to the spread of AMR, posing a serious public health risk [5]. However, AMR is not confined to humans only. The spread of AMR bacteria and genes across ecosystems can take place through various pathways connecting humans, animals, and the environment [6].

Therefore, the One Health approach is necessary to address the rise of AMR due to the interconnection between humans, animals, plants, and the environment. The strategic objectives of the One Health Priority Research Agenda include: (1) improving the understanding of key drivers of AMR transmission and impact, (2) strengthening evidence for interventions through sustainable and multisectoral surveillance, and (3) advocating for AMR mitigation and informing policymaking by assessing the social and economic impacts of AMR on health. In addition, five key areas of concern have been identified: transmission, integrated surveillance, interventions, behavioral insights, and economics and policies. These are emphasized to be examined across three cross‑cutting themes: gender, vulnerability, and sustainability [7].

The concept of vulnerability in AMR encompasses economically, socially, or otherwise marginalized populations. These populations are more susceptible to harboring resistant microorganisms, acquiring resistant infections, and experiencing economic hardship indirectly caused by AMR. Among the vulnerabilities, the document “WHO global research priorities for antimicrobial resistance in human health” specifically prompts investigation of AMR in bacteria prevalence among migrants [8]. Migrants are one of the most vulnerable representative groups for global health. The change of environment, language barriers, discrimination, and poor income concur to challenge access and retention into the healthcare system for the migrant patients in the host country [9].

Furthermore, similar to AMR, migration is a complex phenomenon, and a one‑dimensional approach would limit its understanding. Various types of migrants can be identified (Supplementary Table 1). Migration flows are typically from low‑ and middle‑income countries to high‑income countries [10], even though this statement is not exhaustive (Box 1). Contrary to the assumptions of many healthcare professionals in high‑income countries, the burden of AMR is extremely high in low‑ and middle‑income countries. At the WHO regional level, the highest death rates attributable to AMR are found in western Sub‑Saharan Africa. Low‑income countries are disproportionately affected by AMR due to factors such as a high burden of infectious diseases and limited access to quality healthcare. Inadequate infrastructure, such as poor laboratory facilities and lack of access to clean water and sanitation, further exacerbates the issue, impeding the development of regulatory frameworks, including infection control policies and antimicrobial [11, 12].

Beyond the prevalence of AMR in origin countries, two other important aspects should be considered: (1) each migrant’s history exposes them to different AMR risks and (2) AMR also represents a major problem in host countries. Considering the European context, the predicted occurrence of deaths associated with AMR in 2050 is higher than the overall trend (90.5 versus 87.7 per 100,000), as well as AMR‑associated disability‑adjusted life years (DALYs) count overcomes the number referred to high income countries (HICs) in general. In this context, we aimed to conduct a review to provide a comprehensive evaluation of the determinants involved in AMR carriage and acquisition risk among migrants, current data on the topic, surveillance strategies, and strategic policies implemented to mitigate the impact of AMR on migrants’ health. The focus of this review is to examine the role of migration in the AMR phenomenon. Consequently, we concentrated on the microorganisms mentioned above for two reasons: (1) these pathogens significantly impact the epidemiology of infections associated with medical care, necessitating health institutions to revise general policies and (2) their spread is closely linked to colonized individuals and their living conditions, which is one of the most critical issues in migrant health.

**Box 1. Do migrants really move mostly from LMICs and MICs to HICs?**

Migration is not a single‑step process, and the definition of hosting countries must consider that the migration route can last for years and that individuals move through different countries. Furthermore, the definition of migrants (e.g., refugees and economic migrants) is always challenging, and overlapping conditions are often present. For this reason, some data may question the idea that individuals move from low‑middle income countries (LMICs) to high income countries (HIC) in the highest numbers. As a matter of fact, 75% of refugees are hosted in LMICs or middle income countries (MICs) (UNHCR, 2023) [13]. Nevertheless, two observations are required: (1) refugees account for about 40% of the total 281 million migrants, and (2) internally displaced people (IDPs) represent 60% of refugees, with this phenomenon being almost exclusively present in resource‑limited countries (IOM, 2024) [10]. Hence, it can be observed that, in general, the migration flow goes from resource‑limited countries to high‑resource countries (IOM, 2024) [10]. To support this, it can be observed that remittance flows are mainly directed from HICs to LMICs and MICs (IOM, 2024). This consideration influenced our review, and some disclosures should be made. First, the studies included are mostly set in HICs. This may be due to the higher resources required to perform AMR studies, resulting in less literature about AMR and migration within the LMIC and MIC context. Second, IDPs are almost not mentioned. This category is mainly encountered in LMICs and MICs, as these countries are the most exposed to catastrophic events [14]. Additionally, their condition predisposes them to a different kind of resettlement process. For this reason, we believe that an evaluation of AMR within IDPs would require a specific research design. Some authors who focus on the forcibly displaced stressed the difficulty to distinguish between internationally displaced people and internally displaced people [15].

## 2. Methods

### 2.1 Search strategy

A comprehensive literature search was conducted on 21 September 2024, using three primary databases: PubMed, Scopus, and Embase. The search terms employed aimed to capture relevant studies related to the intersection of migration and antimicrobial resistance (AMR). The following search string was used for Pubmed: ((“Antibiotic resistance”) OR (“Antimicrobial resistance”) OR (“AMR”) OR (“Multi‑drug resistant”) OR (“MDR”)) AND ((“Migrant”) OR (“Refugees”) OR (“Asylum seekers”) OR (“Internally displaced people”)). This string was later adapted for Web of Science and Embase.

This combination of terms was designed to retrieve studies discussing the global concern of AMR, with particular focus on migrants’ population, refugees, asylum seekers, and internally displaced people. Additional terms, including pathogen‑specific acronyms, for example methicillin‑resistant S. aureus (MRSA), Klebsiella producing carbapenemase (KPC), extended spectrum beta‑lactamase (ESBL), and so on) and relevant antibiotics were employed in PubMed searches for further specificity regarding AMR‑related data.

### 2.2 Study selection and inclusion criteria

This literature review aims to provide an overview of the interaction between migration and AMR. Therefore, our review used a hierarchical approach, preferring, where possible, systematic reviews and meta‑analyses of intervention studies followed by the secondary synthesis of observational studies. However, if not available, descriptive reports, narrative reviews, and observational studies were also considered when they provided novel insights or addressed gaps in the current understanding of migration and AMR.

To define migrants, official documents from United Nations (UN)‑endorsed institutions, as well as high‑ranked publications, were consulted. Supplementary Table 1 outlines a suggested list of definitions based on this review. Due to the difference of the burden of infections, most of the literature and, thus, our review focuses on AMR in bacteria.

### 2.3 Screening process

No specific time restriction was applied in the search; however, preference was given to the most recent literature, particularly works published after 2000. Guidelines and official documents have been actively searched on the web. Titles, abstracts, and full texts were reviewed to ensure relevance to the topic of healthcare provision for migrants and their relationship with AMR. Studies were included based on their thematic focus and contributions to understanding healthcare interventions, AMR transmission risks, and public health challenges unique to migrant populations. No formal quality assessment was performed due to the narrative nature of the review.

## 3. Result

### 3.1 Prevalence of antibiotic resistance in migrants

Data about colonization or infection by AMR pathogens among the migrant population are an ongoing hot topic in ‘Infectious Diseases and Global Health’ discourse. The quality of data may be partly attributable to poor surveillance in migrants’ origin countries, as well as among migrant populations in host countries [16]. In recent years, migrants arriving in Europe are mostly refugees from countries whose data on antibiotic‑resistant organisms are not available. Nevertheless, a high incidence of AMR has been described in neighboring countries, for which data have been published [17].

According to a large meta‑analysis by Chukwudile et al. conducted in 21 studies, including 14,168 migrants in Europe, the prevalence of any detected AMR carriage or infection among all migrants was 28% [18]. Forced migrants (refugees, asylum seekers, and migrant children) represented 43% of total population and presented an analogue prevalence rate, while other migrants (worker, student, family‑reunited, and so on) showed 32% prevalence rate in the pooled analysis. Interestingly, community settings with high numbers of forced migrants, such as camps or transit and detention centers, had even higher prevalence than hospital settings (41%), being gram‑negative higher among other migrants and MRSA higher among forced migrants [18]. Those data showed an increasing trend with respect to the 2018 meta‑analysis by Nellum L.B. [16]. Furthermore, the data also increased for AMR‑related infection, reporting 3.0% in 2017 and 41% in 2024 [16, 18]. Interestingly, the data about AMR colonization are lower (22%) [18], and in the meta‑analysis in 2018, the percentage of forced migrants was consistently higher (77%).

Nevertheless, the authors do not compare data among migrants and the general population. In 2022, a highly‑cited systematic review reported an estimated prevalence of MRSA for each European country: the percentages show high heterogeneity, from less than 5% in Scandinavian countries to slightly less than 40% in Portugal [19]. Again, the authors report country‑specific data for third‑generation cephalosporin‑resistant *Escherichia coli* and *Klebsiella pneumonia*, fluoroquinolone‑resistant *E. coli*, carbapenem‑resistant *Acinetobacter baumanni* and *K. pneumonia*. These data show highly varied prevalence levels, ranging from less than 5% to slightly less than 50% or even more than 80% for *A. baumanni*, according to the country [19].

By comparing these data, it can be assumed that migrants are prone to being carriers of AMR. In this regard, many studies directly analyze differences in data between migrants and natives.

In a recent study of 2022 including clinical samples from 37,276 individuals in Denmark, family‑reunited migrants and refugees were seen to have higher levels of ciprofloxacin‑resistant *Enterobacterales* compared with nonmigrants [20]. Furthermore, in this study, *Staphylococcus aureus* was the most frequent pathogen isolated in both migrants and nonmigrants’ groups; however migrants, especially female migrants, had notably higher odds of MRSA compared with nonmigrants [20]. Nonetheless, both MRSA and ESBL‑producing bacteria prevalence rates resulted lower than those in previous European studies [21]. This can be explained by the fact that the Danish study enrolled migrants who have been residents in Denmark for a median duration of nearly 10 years with a possible decline of AMR in bacteria overtime [20]. This confirms that overcrowded living conditions, limited access to healthcare, and inadequate sanitation in refugee camps or migrant shelters are all determinants of spreading AMR in bacteria.

A study conducted in the Netherlands among asylum seekers found MRSA more frequently in clinical cultures than in screening cultures, indicating that MRSA strains in this population may be more pathogenic, leading to infections that are more likely to be identified through clinical testing due to treatment failures. The same study included 58,748 strains positive for Enterobacteriaceae (78.1% obtained from urine samples). Multidrug‑resistant Enterobacteriaceae (MDRE) were found in 38 (17.7%) samples among asylum seekers and 2,554 (4.3%) from general population patients [22].

In 2019, Reinheimer C. et al. performed a retrospective case–control study to assess whether intensive care unit (ICU) patients coming from refugee facilities in Germany presented higher prevalence of MDR gram‑negative bacteria (MDRGN) or MRSA colonization than natives. Both the prevalence was higher and associated with the time period from arrivals. Being in Germany for less than 3 months was a risk factor for colonization. Equally, a gradual decline was observed until 18 months, when MDRGN substantially reached native patients’ rate, but MRSA still persisted higher [23]. In 2022, Creutz et al. performed both the genotype identification of *S. aureus* resistance and the antibiotic susceptibility testing (AST) among healthy refugees. One of the four MRSA‑colonized strains showed the same profile responsible for several documented outbreaks in Denmark, which involved both migrants and travelers [24, 25].

Some studies also tried to assess the specific effect of countries of origin and AMR colonization rate. In 2018, Aro T. et al. described MDRO screening results among the refugee population in Helsinki hospital. The prevalence was 32.9% for ESBL‑producing Enterobacterales (ESBL‑PE), 21.3% for MRSA, 0.7% carbapenemase‑producing Enterobacterales (CPE), 0.4% multiresistant *Pseudomonas aeruginosa* (MRPA) and 0.4% multiresistant *Acinetobacter baumannii* (MRAB). Interestingly, the geographical origin was independently associated just for ESBL‑PE with North Africa and Middle East African and Asian patients [26]. In 2017, Piso R.J. et al. reported a high prevalence of MRSA and ESBL in some Swiss refugee facilities with a major correlation of ESBL colonization with origin from Middle East countries [27].

In 2018, Ciccozzi et al. performed swab screening for MDRO in an Italian refugee facility, finding a high prevalence of 34.7% and 22.4% for MRSA and ESBL‑PE, respectively [28]. Equally, a 2023 study examining 3,960 nasal swab samples from Syrian refugees and resident Turkish population revealed significantly higher rates of antibiotic resistance among the refugees. Specifically, the prevalence of MRSA was higher among Syrian refugees. Additionally, the study found that 17.9% of the stool samples from Syrian refugees were positive for ESBL isolates, compared with 14.3% in the local Turkish population (*p* = 0.041), with no relationship found between ESBL positivity and previous antibiotic use or hospitalization. Notably, 62.9% of the AmpC‑positive isolates were from Syrian refugees [29]. By the way, the Syrian migrant population is a good example of country‑targeted investigation: a review by Osman M. et al. collected all works about Syrian refugees in host countries and commented on the prewar and on the ongoing Syrian context [30].

Another Danish study analyzing data from 14,561 urine samples between 2000 and 2015 found that antibiotic resistance was generally higher in *E. coli *isolates from migrants compared with nonmigrants. Specifically, resistance to sulfamethoxazole‑trimethoprim was found in 34.3% of *E. coli* isolates among migrants, compared with a lower percentage in nonmigrants [31].

In 2016, Heudorf U. et al. recorded the prevalence of MRSA and MRGN among refugees in hospital settings in Germany [32]. The prevalence was 9.8% for MRSA and 23% for MRGN (including ESBL and carbapenem resistant). This report highlights the heterogeneity of findings comparing its data with previous data both from hospital settings [22, 33–36] and refugee facilities [37, 38]. Notably, the article compares the prevalence of MDRO with data from some risk factor categories for AMR, such as patients undergoing hemodialysis (HD) and patients receiving nursing for elderly assistance. The prevalence of MRSA among refugees was higher than the one among these two categories, while the prevalence of MRGN was higher than HD but in some conditions lower in elderly patients receiving nursing [32].

As a major hot topic in AMR being the difference between clinical infections and colonizations, we differentiate the origin of the isolates from the studies included in our review in Table 1.

**Table 1:** Origin of isolates among studies of the review.

CLINICAL SAMPLES	COLONIZATION SCREENING	MIXED	MIXED + ENVIRONMENTAL TEST	UNSPECIFIED
Nielsen [20] Sloth [31]	Angeletti [38] Ciccozzi [28] Creutz [24] Heudorf [21] Heudorf [32] Heudorf [37] Piso [27] Reinheimer [33] Reinheimer [23] Steger [34] Yıldız [29]	Aro [26] Ravensbergen [35] Ravensbergen [22]	Møller [25]	Peretz [36]

### 3.2 Pathogen‑specific consideration

This review focuses on bacteria involved in healthcare‑associated infections or common infections caused by bacteria that are not exclusively pathogenic but can also be part of the normal microbiome. Further considerations are warranted for pathogens associated with specific conditions. In 2020, Hernando Rovirola C. et al. analyzed data from European Gonococcal Antimicrobial Surveillance Programme (Euro‑GASP) in the years 2010–2014 and observed that azithromycin resistance, ciprofloxacin resistance, and decreased susceptibility to ceftriaxone did not differ between natives and foreign born; conversely, a lower rate of cefixime resistance was recorded among migrants. Nevertheless, an association between being born in a WHO Eastern Mediterranean Region and non‑EU/EEA WHO European countries and AMR isolates was found, and the increased rate of migrants contracting the infection abroad versus reporting countries highlights the important role of migration in the spread of resistance in a sexually transmitted infection [39].

AMR also affects syphilis. Although the most updated institutional report raised the issue [40, 41], no epidemiological data about drug resistance are included, but the importance of monitoring through molecular diagnostics is confirmed. Still, a meta‑analysis in 2022 reports the prevalence of A2058G and A2059G mutations associated with macrolides and tetracycline resistance, differentiating between high‑ and low‑income countries [42]. The rate varies from about 90% in China to a range between 0% and 22% in African countries. Although data related to the migrant population are not available, the correlation with rate in countries of origin suggest that migration could play a role in spread of resistance to anti‑syphilis treatment.

Last but not least, tuberculosis (TB) is one of the most discussed topics for the association between drug resistance and migration. Many countries of origin are included in the top ten of countries with a high burden of multidrug‑resistant TB [43], and the challenging treatment, along with precarious life condition associated with migration status, increases the risk of poor adherence to the treatment, which is one of the most important predisposing factor for resistance development [44].

However, these specific conditions are beyond the scope of this study, and we refer a full and detailed discussion to other works.

### 3.3 Spatial distribution of migrants with AMR

In Figure 1a and b data about the prevalence of gram‑positive (*S. aureus*) and gram‑negative bacteria in the countries from the works are exposed in our study. Interestingly, Germany, Italy, the Netherlands, Switzerland, and Turkey rank among the top ten European countries hosting the highest foreign‑born or refugee/asylum seeker population per citizen/in absolute numbers [45, 46], whereas a study from Finland is included. Our study is a literature review, which leads to limitations, which could be overcome by systematic review study design. For this reason, we refer to the meta‑analysis by Chukwudile B. et al. for a more comprehensive discussion about prevalence of AMR based on host countries and origin regions [18]. Here, some studies from France, Spain, and Greece are also included, as these countries are of great importance for the density of the migrant population and for arrivals.

**Figure 1a:**
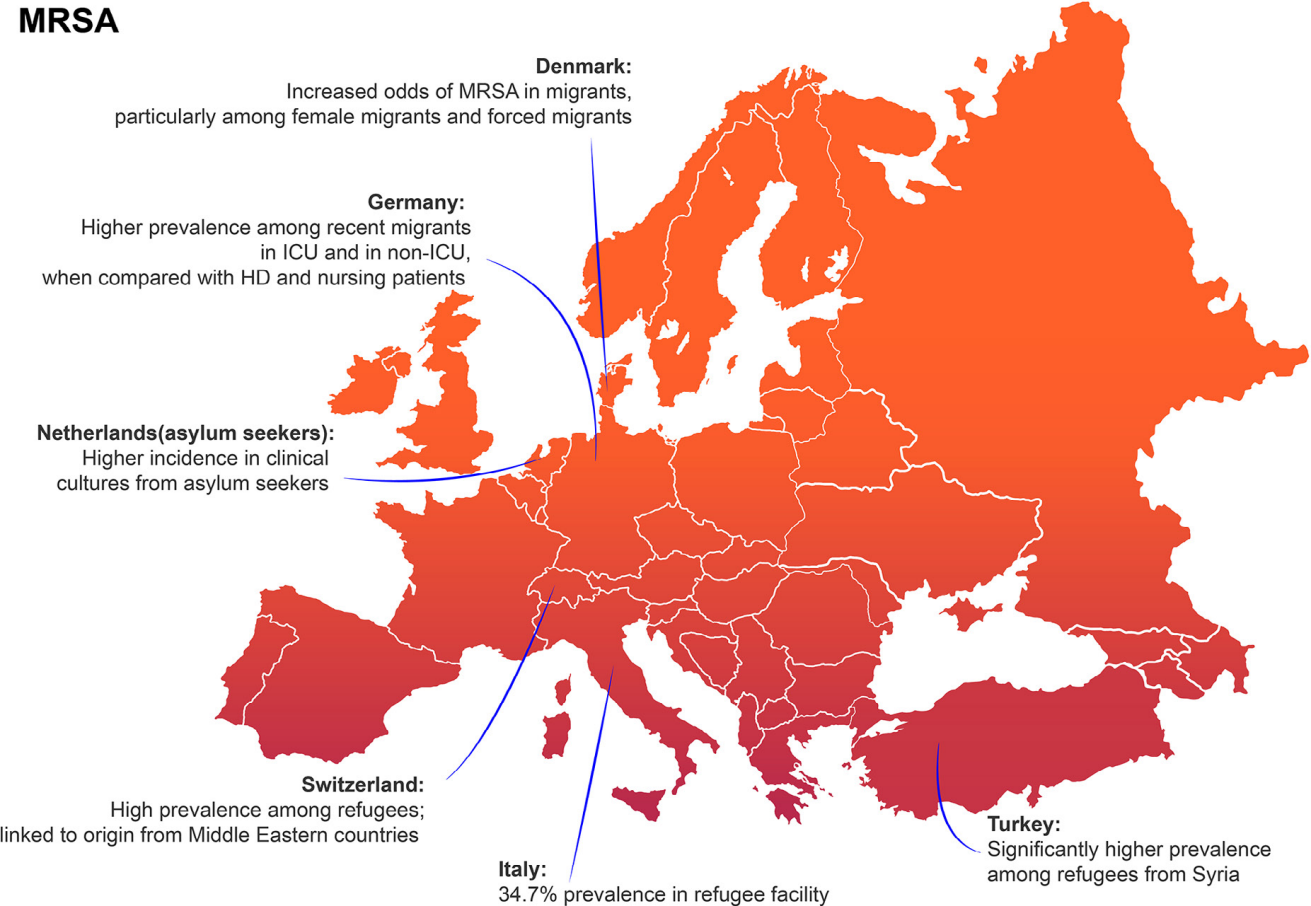
Recap of evidences about MRSA and migration in Europe. MRSA, methicillin‑resistant *Staphylococcus aureus*; ICU, intensive care unit; HD, hemodialysis.

**Figure 1b:**
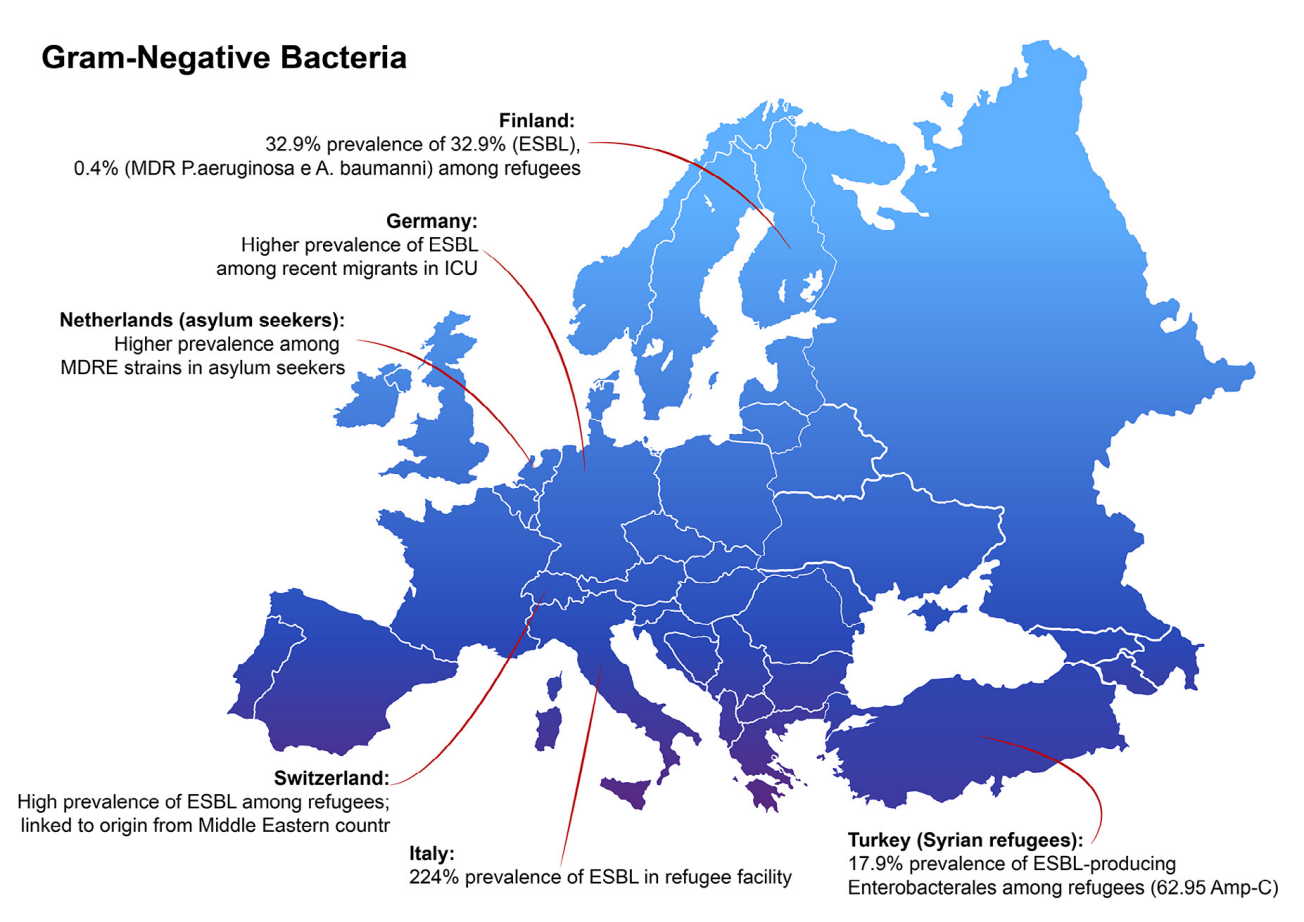
Recap of evidences about gram negative pathogen and migration in Europe. ESBL, extended spectrum beta‑lactamase; MDR, multidrug resistant; ICU, intensive care unit; MDRE, multidrug‑resistant Enterobacteriaceae.

Nevertheless, some considerations should be made about the demographic aspect of the migration phenomenon in Europe. European law is a composite system, including a mix of international and national law with additional mutual agreement between EU and non‑EU nations. According to the Dublin Agreement, each person entering EU territory is bound to the country of arrival for an asylum‑seeking procedure, while the Schengen Agreement allows free movements through participating countries. Also, many countries have specific agreements related to former colonies [47]. Therefore, any assessment of AMR concerning the migrant population should take into account that movements of migrants across national borders can influence evaluations based solely on origin and host countries.

Furthermore, European countries do not share a homogeneous definition of migrants nor a uniform health access bureaucracy for foreign‑born individuals and refugees. These two aspects negatively impact the production of literature and the reporting of issues related to migration [48].

Additionally, it should be remarked that the works included in our review are mainly from European countries, as the current topic is the object of publications mostly in this setting. Further considerations have been made in Box 2.

**Box 2. Other contexts**

The search strategy of our review led to an overwhelming representation of European studies. We tried to widen our search, but scarce results were found. A meta‑analysis in China reports that migration status is often included among risk factors for AMR development in healthcare settings, even though no specific data could be included in the statistic [49]. Most other works responding to multidrug resistance in China focused on MDR‑TB. In Nepal, a study devised a mathematical model to investigate the potential epidemic drivers of typhoid in Kathmandu, reporting a correlation between the increase of cases and of fluoroquinolone‑resistant prevalence and internal migration [50]. An increased rate of MRSA has been described among Bhutanese refugees in Nepal, both in Nepal and after resettlement in the USA [51]. Still, the contemporaneity of the issue promises an uprising publishing activities beyond European borders, as shown by the recent publication of a meta‑analysis about AMR among refugees and asylum seekers by Hermens E.D. et al. [52]. Beyond basically confirming AMR increased risk for asylum seekers and refugees, this work highlights that the main publishing activity takes place in Europe, being only 8 out of 41 the non‑European articles included in the systematic review.

### 3.4 Risk factors for antibiotic resistance among migrants

The migration status involves several features, which are thought to be involved in the AMR colonization risk, as migration is generally related with low socioeconomic conditions.

In 2018, a meta‑analysis by Alividza V. et al. explored single factors that characterize poor living conditions such as housing, low income and income inequality, education level, water and sanitation, and social deprivation and their correlation with AMR [53]. Interestingly, just one among the included studies explicitly assumed migration status as socioeconomic variables: this study could not find any correlation with penicillin‑ nonsusceptible *Streptococcus pneumoniae *isolation in screening/clinical samples [54].

Nevertheless, other studies describe that the dwelling conditions related to migration (both in transit status and in life in host countries) facilitate the AMR acquisition. In a meta‑analysis by Nellum L.B. et al., the prevalence of AMR was higher in high‑migrant community settings compared with hospital‑setting findings and with the data concerning all migrants [16]. The transit and staying in refugee facilities has been considered a risk for two reasons. First, they are often characterized by poor Water, Sanitation, and Hygiene WASH and dwelling, with suboptimal hygiene conditions and overcrowding [55, 56]. Second, difficult access to health facilities and low quality of assistance are often encountered in migration centers. Some authors observed that poor health assistance influences the antibiotic consumption among refugees, as physicians tend to overprescribed antibiotics to compensate for the language barrier [57] and that migrants practice self‑medication with antibiotics as compensation behavior for precarious life condition perception [58–60]. To confirm that, Ladines‑Lim J. et al. describe language skill as a protective factor against AMR among migrants [61].

Additionally, the rate of antibiotic consumption in the country of origin should be considered. According to an evaluation performed by Browne A.J. in 2021, LMICs present a lower consumption rate of antibiotics than HICs. Nevertheless, the trend follows different patterns; in LMICs, consumption is increasing, while in HICs persistence is stable [62]. As AMR prevalence is higher in LMICs than HICs [19] and LMICs are more affected than HICs in terms of disability‑adjusted life‑years (DALYs) and mortality [1], it can be suggested that the use of antibiotics is inappropriate both for dosage and for indications.

Last but not least, data about gender differences should be commented on. Unfortunately, there is a evident research gap that masks the results about a possible intersection between AMR, migration, and gender. Due to the complexity of the issue, we discuss it separately (Box 3).

**Box 3. Gender disparities**

Limited data are available on gender disparities at the intersection of AMR and migrant health research. The two most recent and comprehensive meta‑analyses [16, 18] fail to provide a targeted gender analysis, as do the other papers included in our review. Being a woman adds additional barriers through a nested box mechanism, where gender‑based discrimination is compounded by migrant status. A scoping review on maternal health underscores this phenomenon, emphasizing how pregnancy‑related care serves as a bridge for women’s access to healthcare [63]. Nevertheless, the condition of being a racialized woman is exposed to lower quality of access to services [63]. Additionally, female‑focused research is often concentrated on sex work, a major topic in gender studies. A systematic review by McBride B. et al. highlights that criminalization and restricted healthcare access disproportionately affect migrant women and transgender individuals engaged in sex work [64]. While these studies explicitly address gender disparities, two key considerations must be noted. First, they do not directly address AMR, evidencing the research gap and gender disparities in the field. Second, narrowing gender research to these specific fields risks reinforcing stereotypes and oversimplifying the gender‑based impact on medical outcomes. Gender clearly plays a crucial role in sex work for representation and maternal healthcare. Maternal healthcare also indirectly influences AMR prevention in children. A health literacy survey on AMR in the USA found that female gender was independently associated with higher health literacy, likely due to the role of women as primary child caregivers in their countries of origin, reinforced by WHO‑led campaigns [61]. Despite these insights, the overall burden of discrimination faced by migrant women in daily life should take priority and include medical issues beyond sexual and reproductive health. Given that AMR research is still evolving, it presents a unique opportunity for gender‑based studies to introduce a new research methodology and expand beyond traditional gendered topics.

## 4. Discussion

### 4.1 International mobility and AMR

Interestingly, some studies hint that the movement of migrants is not the greatest cause for AMR spread worldwide. International travelers seem to play a more important role in carrying AMR pathogens from nations to nations [65]. Medical tourism or job travel facilitate the spread of peculiar pathogens from developing countries, such as bacteria related with diarrheal syndrome (*Salmonella spp*. and* Campylobacter spp*.). This enhances the need for a pretravel vaccine program, to reduce both the colonization acquisition and the access to health facilities in countries at risk for AMR [66]. To confirm this statement, a study by Monsálvez V. et al., among 122 long‑term travelers and recently arrived migrants, found that only a history of hospital access and chronic diseases were recognized as risk factors for AMR acquisition [67]. Furthermore, international travels introduce specific genes in other environments, which consequently start being translocated to other bacteria (through horizontal gene transfer of AMR determinants). This is the example of the NDM1 pandemic and other ESBL genes [65, 68].

Curiously, although Frost I. et al. state that colonized travelers tend to revert their AMR colonization after some months [65], some data suggest different conclusions for long‑term migrants [69]. For instance, Nielsen R.T. et al. report higher prevalence of AMR among family‑reunified migrants and refugees in Denmark compared with natives [110]. Nevertheless, as previously mentioned, data from a Nielsen R.T. et al. study in 2022 highlights that AMR colonization among migrants decreases year by year after arrival [20]. In our review, we mentioned other studies which try to explore the relation between time from arrival and AMR colonization [20, 23]. Data could somehow seem not conclusive. Still, this could support the assumption that country of origin is not the only risk factor for AMR among migrants but life conditions and social inclusion concur in a consistent way. Nevertheless, Nellum L.B. et al., discourage the correlation between AMR colonization risk among the migrant population and spread among native people [16].

### 4.2 Migration‑related phenomenon and AMR

Further considerations could be made about three hot topics related to migration: conflict‑related refugee crisis, climate change‑driven migration, and labor conditions of migrant workers in host countries. Conflicts and humanitarian crises, which drive migratory movements globally and lead to significant and often violent population displacements, can have a distinctive and multidimensional impact on the health system of the countries by depleting not only surveillance systems and infection control measures but also healthcare assistance.

A comprehensive understanding of the links between war and AMR and its global implications is potentially crucial; as studies conducted in Syria have shown, the adverse conditions established as a consequence of these prolonged conflicts (crowding, poor ventilation, inadequate shelter, malnutrition, inappropriate surveillance of antimicrobial use, collapsed infrastructure and healthcare facilities, lack of sanitation within the camps, and limited access to healthcare and immunization) is an important challenge [70]. Additional possible correlations have emerged from recent evidence on the Iraq War, highlighted by alarming data on the increased resistance of Acinetobacter caused by environmental contamination with heavy metal (such as lead, mercury, and copper) due to debris and gun shrapnel dispersal into the soil [71]. In more recent years, the Russian–Ukrainian war concerns epidemiologists for the great burden of AMR observed in military hospitals [72]. As the wars disrupt the routine facilities of a country, it is easy to understand that civilian Ukrainian refugees cannot be spared from AMR concerns [73].

Beyond conflicts, the intersection between climate change and AMR may involve migration and require comprehensive approaches that address environmental and health determinants. Climate‑change‑induced events such as extreme weather events, sea‑level rise, and droughts can force people to migrate and displacement frequently results in overcrowded living conditions, limited access to clean water and sanitation, and constrained healthcare resources, increasing the risk of infectious diseases and the inappropriate use of antibiotics, which can contribute to AMR [74]. Additionally, migrants often work in agriculture, where antimicrobials are commonly used in livestock farming. Inadequate regulation of antibiotic use in agriculture can facilitate the transmission of resistant bacteria through food chains, thereby increasing the risk of AMR within migrant communities [74].

### 4.3 One health perspective

The issue of AMR cannot be fully addressed without considering antimicrobial use in the animal industry. In 2017, an estimated 73% of global antimicrobial consumption was for animal use, with an anticipated increase of about 8% from 2020 to 2030 [75]. In livestock systems, the interspecies transmission of AMR—from humans to animals—occurs through both production practices and consumption [76]. AMR acquisition can result from both direct pathogen transmission and horizontal gene transfer, with Enterobacterales, Staphylococci, and Enterococci being key pathogens of concern, although all ESKAPE bacteria should be considered [76].

From this perspective, the interconnected factors of AMR linked to migration and the food industry must be considered on two fronts. First, the use of antibiotics in animals in the countries and regions of origin must be evaluated. Asia has the highest level of antibiotic consumption for animal use, with India and China among the top five consumers in 2020 (as well as Brazil), and Africa also shows an increasing trend over the decade, although accounting for less than 1% of global animal antibiotic consumption in 2020 [77]. Second, occupational exposure to AMR poses a significant risk, particularly for migrant workers. The European Commission’s 2023 report on social protection for seasonal workers in the agriculture and food industries underscores concerns about suboptimal working conditions for this category [45]. Notably, migrants are about fivefold more represented in these jobs than the nonmigrant population [78]. However, occupational and consumption‑related AMR risks should not be regarded as geographically isolated. For instance, while the USA and Australia were also among the top five consumers of veterinary antibiotics in 2020, and European countries such as Italy and Germany have also reported areas of high antimicrobial use [77].

Given these factors, a One Health perspective emerges as essential for addressing the overlapping impacts of migration and AMR effectively, with human use of antimicrobial drugs being only partially responsible for the development of resistances.

### 4.4 Challenges in AMR containment: strategies and intervention

**Figure 2:**
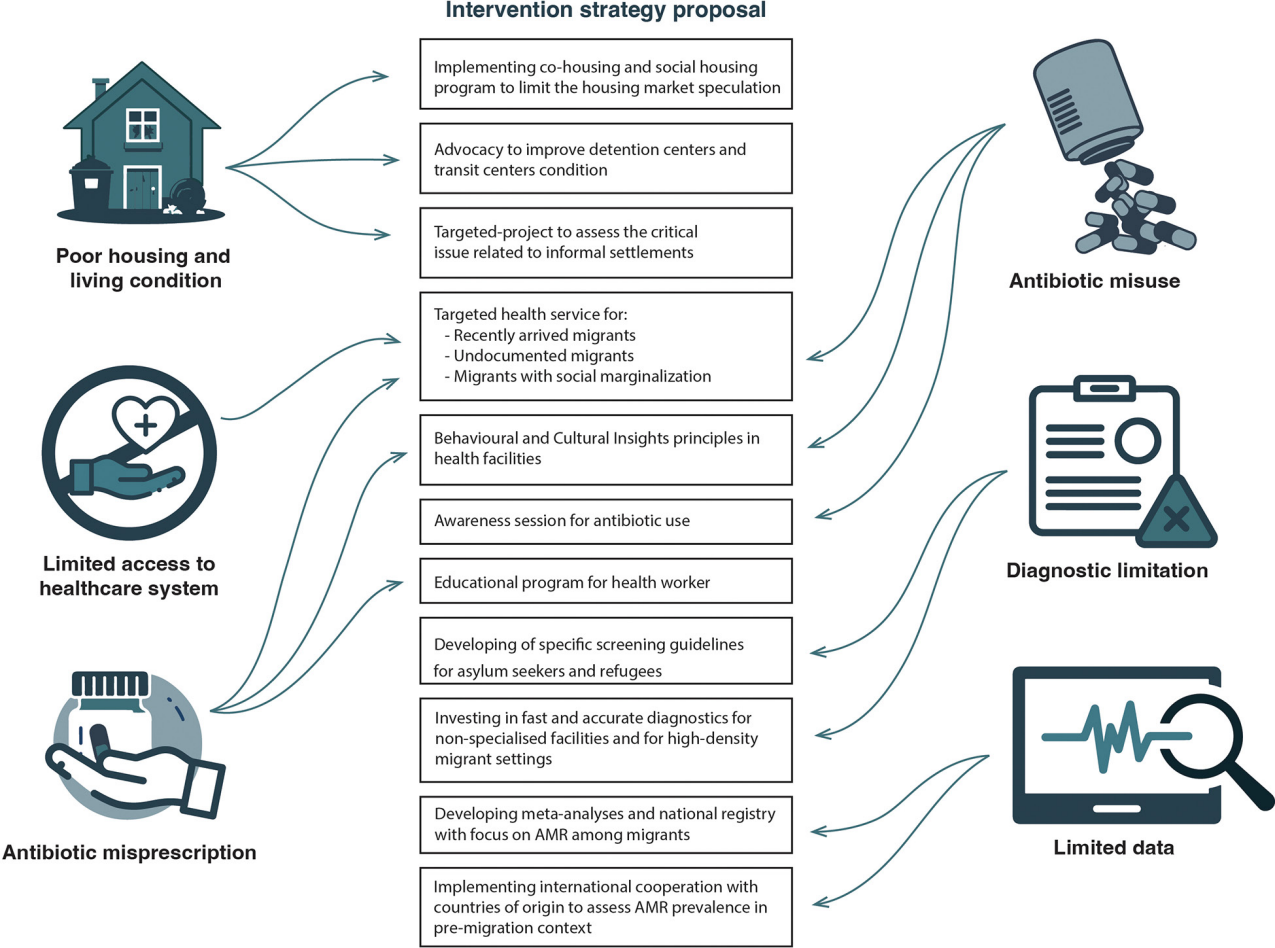
Five key points can be used to summarize the intervention proposal, covering the various aspects involved in the AMR phenomenon in migrant populations.

As previously discussed, the approach to AMR in migrants is a challenging hot topic in public health. Both AMR and migrant health represent global challenges that require concerted efforts and cooperation among countries and organizations. A wide range of disciplines (such as health sciences, epidemiology, microbiology, economic, social and environmental sciences, ethnography, and so on) are involved in these issues. First, antibiotics represent a part of routinary life worldwide and for this reason each individual approaches their use with a cultural‑specific perspective depending on the country of origin [79]. A population‑based survey in Denmark explored the role of ethnicity in antimicrobial knowledge and use by interviewing more than 20,000 people. All the people included in the migrant population result in having less knowledge about antibiotics compared with natives, with a consequential incorrect use [80]. Still, the actual odds for antibiotic prescription were not uniform among all the countries of origin. One of the most illustrative issues of a culture‑based approach to antibiotics is that, in many countries, antimicrobials are accessible without a medical prescription, which has been associated with a higher prevalence of AMR [81]. Second, the health operators could be unprepared to face the AMR among migrants. On one hand, unusual bacteria have been encountered in screening of refugees [38], whose virulence is hardly known among clinicians. On the other hand, specific outbreaks among refugee facilities could require second level diagnostic tools, which are not available in any setting [82]. These factors combine with the previously discussed language barrier and precarious access to the health system for migrants [83]. Third, the costs for AMR diagnostics burden the resources for health assistance in the migration management. If syndromic surveillance could play a significant role in health management in refugee facilities [84], the molecular diagnostic or the AST tool increases the costs and complicates all the procedures. Consequently, effective collaboration among numerous stakeholders is essential. Coping with AMR in the context of the migrants’ health, it is imperative to identify and mitigate its primary drivers. In this regard, we proposed four crucial topics: (1) improvement of housing and sanitary conditions, (2) ensure migrants’ access to healthcare services, (3) promotion of appropriate use of antibiotics by both patients and healthcare providers and (4) enhancement of microbiological surveillance and diagnostics. In the author’s opinion, these are the pillars for an interventional strategy proposal ( Figure 2).

#### 4.4.1 Improvement of housing and living conditions

Overcrowding and substandard housing conditions and homelessness are notably more common among the migrant population than the native population throughout Europe, even a significant time after arrival, possibly due to limited knowledge of the housing market, language barriers, discrimination, and low income [85]. Therefore, although many countries have implemented innovative strategies to address the issue of migrants’ living and medium and long‑term housing conditions (such as partnership‑based approaches, cohousing, accompanying housing with employment and social services, and so on) [86, 87], improving the housing conditions of migrants is still an ongoing process on the upcoming Agenda of the European Commission [88]. Notably, the migration journey represents a consistent part of exposure to overcrowding and poor living conditions for migrants. The most critical living conditions predisposed to AMR are found in detention centers, transit centers, reception facilities, and informal settlements. In these settings, minimum water, sanitation, and hygiene standards are often not met, due to a lack of space, clean water, food, and essential care [56, 89]. For this reason, international coordination and interventions should aim to ensure basic hygiene and sanitation standards at each point of arrival and transit along migration routes. Particularly, informal migrant settlements should be urgently prioritized for the complexity of their context [90].

#### 4.4.2 Equitable access to healthcare services for migrants in host countries

As highlighted in the WHO’s 2022 report *“Capturing the evidence on access to essential antibiotics in refugee and migrant populations,”* the barriers between migrants and healthcare services are multifaceted and complex, encompassing factors from both health system side (long waiting times, availability of services, costs, and so on) and migrant’s side (fear of deportation, preference for self‑medication and for products from the country of origin, and so on) [91]. The identification of these barriers to access to formal care at the local, national, and transnational level is crucial to guide health policies aimed at eliminating them through targeted interventions, such as the elimination of legal status as a criterion for receiving healthcare, the introduction of interpreters or bilingual staff to improve language accessibility, training programs for health staff to improve migrant‑sensitive cultural competences, improving migrants’ knowledge of rights, and procedures for accessing health services, ensuring the affordability of the services [91].

As we previously debated, migration is a condition predisposing to vulnerability. Hence, some experiences show that outreach programs are sometimes required to link migrants, especially the ones living in most precarious conditions, to the health system, even when access to health facilities is formally guaranteed [92]. The outreach project strategy has been largely employed also outside the urban context. For decades, the agricultural migrant worker has represented a hot topic for their intrinsic precarious condition, depending on high internal mobility and hard‑to‑reach living locations [93]. The phenomenon and its implications in the management of access to health are so relevant that in 1962, US President John F. Kennedy included it in the Migrant Health Act, and today, major international governmental institutions continue to include it in their programs [94, 95]. However, as authors, we emphasize that the misuse of outreach programs, especially when provided by noninstitutional organizations, can foster the marginalization of vulnerable populations, disincentivizing integration into the formal health care system. Nevertheless, outreach programs for agricultural workers should be combined with fair trade evaluations of job contracts and working conditions, as labor exploitation is a negative determinant of health. Irregular employment, poor living conditions, and inadequate workplace safety often act as cofactors; therefore, they cannot be addressed as isolated issues [85].

#### 4.4.3 Appropriate use of antibiotics by both patients and healthcare providers

Access to healthcare services and the continuum of care is crucial to minimize misuse and overuse of antimicrobials by migrants and refugees. A recent study conducted among migrants in the US showed that the primary reason for use of unprescribed antibiotics is the lack of access to health care and also the complexity in procedures for accessing the health system services and language barriers [96]. Furthermore, the cultural belief about self‑medication should be considered, as previously discussed in the article [57, 59]. For this reason, educational programs should target migrant communities. Although the Latino community cannot be considered exclusively as a migrant community in US settings, a good example of awareness program is reported by Stockwell M.S. et al.: in this work, parents were aware that antibiotics are inappropriate in the context of upper respiratory infections [97]. Our research among current literature could not find several references for other experience reporting migrant‑targeted educational programs for antimicrobial usage. Nevertheless, specific campaigns or awareness sessions could benefit from use of cultural mediators, as demonstrated by the role of this professional in other health settings for migrants [98]. It is necessary to fill the cultural knowledge gap by collecting reliable data on the habits and beliefs regarding antibiotic use among migrants. These data are crucial to guide tailored educational programs, taking into account the heterogeneity of this population and the cultural differences between ethnic groups. In this regard, WHO, recognizing the importance of the cultural background, beliefs, and deep‑rooted habits of different groups in addressing the issue of antimicrobial resistance, has proposed the use of a Behavioral and Cultural Insights (BCI) [99] approach in national health policies. The BCI approach focuses on understanding the individual and contextual factors that influence antibiotic use. By drawing on various social sciences, it offers a people‑centered approach to policy making. In fact, the cornerstones of the BCI approach lies in the implementation of tailored solutions based on the identification of barriers and drivers of appropriate antibiotic use in specific contexts, the involvement of stakeholders, behaviors analysis, and evidence‑informed interventions to influence and transform behaviors and the evaluation of effectiveness of interventions. Although the members of WHO European Region endorsed BCI as a flagship priority [100], the status report on the use of BCI approach in health policies 2021–2022 revealed that BCI is still underused, underfunded, and under‑resourced among the 48 out of 53 countries of the European region that have provided data [99]. This professional support could be also important in the evaluation part of the awareness session with a pre‑ and postinterview to assess the efficacy of the program [101]. On the healthcare system side, the misuse of antibiotics by migrant patients is also rooted in the excessive prescription by healthcare providers themselves, especially in the context of the healthcare for refugees. The reasons for this excessive prescription can be several, such as linguistic and cultural barriers that make doctor–patient communication difficult, the rapid turnover of healthcare staff and patients in reception, transit, and detention centers, which makes clinical monitoring and eventual delayed prescription of antibiotics after reassessment impossible or even the request by migrant patients for antibiotics [57]. Actually, there are few studies that investigate the causes of overprescription of antibiotics by doctors. Therefore, it is necessary to fill this knowledge gap in the specific contexts of drug prescription to direct training programs aimed at raising awareness and educating healthcare providers with a culture‑sensitive approach.

#### 4.4.4 Microbiological surveillance and implementation of the diagnostics

Beyond education, microbiological surveillance is pivotal to cope with the AMR pandemics, and the migrant context add further challenging factors. Although many studies are available, data are partial and heterogeneous, and it is not known what the real impact of AMR is on this vulnerable population. Therefore, coordinated and systematic microbiological surveillance in places identified as key points for the spread of bacteria among migrants in the migratory route (e.g., refugees camps, transit, and detention and reception centers) is necessary [102]. These data may be useful to identify specific patterns and spreading condition of antimicrobial resistance (spread of fecal bacteria or colonizing the airways and skin, resistance profile to cephalosporins, carbapenems, and so on) associated with specific risk factors in the context of migration (country of origin, migratory route, legal status, stay in refugee camps, and transit and reception centers) and to implement strategies to direct appropriate antibiotic therapies and effective strategies to reduce the spread of AMR among migrants and refugees [103]. Interestingly, a systematic review investigating the screening method for migrants could not record any AMR in bacteria focus [104]. This gap has already been stressed by some authors by proposing to include AMR screening in the service provided by specific refugee‑oriented health facilities [105]. The identification of prevalent resistance patterns and their associated risk factors can guide the development and administration of point‑of‑care tests for use not only in transit and receptions and detention centers but also in general practice clinics. If these tests are rapid, low‑cost, and easy to perform and interpret, they can facilitate the selection of appropriate antibiotic therapies by healthcare providers and prevent the overprescription of antibiotics often observed in migrant patients [106]. Furthermore, the role of technological innovation has also been recognized in the surveillance process in limited source settings. In 2018, a meta‑analysis and systematic reviews reported the cost‑effectiveness of electronic and web implementation for communication between health facilities in a low‑source context. Nevertheless, authors remarked as a limitation the high burden of initial investment. In many countries, these monetary sources are unaffordable, but they could become a target for specific funding by international institutions and stakeholders [107]. However, despite recognizing the strategic role of a comprehensive microbiological surveillance system and POC diagnostics, it is difficult to imagine their implementation in contexts (e.g., Libyan detention centers, Italian hotspots, and so on), where even fundamental standards for health and personal dignity, which remain a priority, are often not met. Contrarily, the development of specific screening guidelines, including AMR, could be considered and Germany provides an example [108]. Nonetheless, Taylor S.L. et al. suggest a comprehensive evaluation that does not focus solely on migration status but includes specific infectious disease considerations to avoid a stigmatizing approach to the issue [109].

## 5. Limitations

Given that this review is primarily narrative in nature, no formal quality assessment was conducted. Moreover, while every effort was made to include high‑quality evidence, the inclusion of descriptive and observational studies means that some findings may be context specific and not generalizable across all migrant populations. Furthermore, the definition of migrant and related categories is not univocal, as previously mentioned; hence, the interpretation of previous studies could not be completely uniformized. Therefore, our review cannot provide a thorough analysis of the various subcategories proposed in Supplementary Table 1. Specifically, the included works that mentioned migrants generally focused on asylum seekers/refugees, while the rest of the papers did not target a specific group. Even when reunited families or long‑term migrants were mentioned, the analysis of their migration background was generic. Consequently, many important categories, such as economic migrants, child migrants, and others, have not been adequately examined.

## 6. Conclusion and Further Considerations

Migration plays a pivotal role in the phenomenon of AMR for several reasons. The living conditions during the journey, along with the precarious and marginalized existence in host countries, often combine with the high prevalence of AMR in the migrants’ countries of origin. Thus, it can be assumed that being a migrant from low‑ and middle‑income countries is a potential risk factor for AMR. Clinicians should consider this in their risk assessments during clinical practice, as migrants appear to have a higher prevalence of AMR compared with the nonmigrant population.

However, several variables need to be taken into account, including specific pathogens, underlying health conditions, geographic origin, social determinants, time since arrival, and many other factors. In light of these complexities, we agree with Taylor S.L. et al. in that the AMR assessment should not be based solely on ethnicity but should instead rely on a multilayered risk evaluation [109].

In the authors’ opinion, the interaction between AMR and migration can be approached from two additional perspectives.

On one hand, researchers and clinicians should focus on: (1) the prevalence of AMR in the countries of origin, (2) quantifying the risks associated with specific conditions that arise during the migration experience, and (3) evaluating specific interventions and validating targeted toolkits.

On the other hand, no studies will effectively address the issue unless policymakers and stakeholders work to eliminate the social determinants of AMR. At the local level, national programs should target the challenging social conditions and barriers to healthcare access by fostering a more inclusive welfare system. Access to healthcare should be regarded as a bidirectional interaction between two or more perspectives and conceptions of health and well‑being. For this reason, the role of cultural mediators should be valued and integrated into healthcare teams to eliminate both cultural and linguistic barriers.

At the international level, institutions should facilitate safer migration processes, and international cooperation should prioritize AMR in both countries of origin and along migration routes. Ultimately, both the One Health perspective and the social science perspective should be adopted as fundamental guiding frameworks, both internationally and in country‑specific policymaking. Collaborative efforts between institutions and stakeholders must transcend national borders, as the spread of AMR arises from interactions among living beings in various forms, which require a comprehensive approach to address effectively. International guidelines, encompassing both medical and social evaluations, international toolkits, resource and data sharing between host and origin countries, the inclusion of a CBI framework with specific health targets in migration policy, and shared data and surveillance between human and animal medicine are all initiatives that could pave the way for effective control of AMR.

In conclusion, we can assert that there is not just one type of migrant, just as there is not a single type of pathogen or antibiotic resistance. If this concept is not fully acknowledged, no intervention will be effective in addressing this complex One Health issue.

## Additional File

The additional file for this article can be found as follows:

Supplementary Table 1. Glossary of Migration and References.
