# Supplementary material for: Antimicrobial Resistance and Migration: Interrelation Between Two Hot Topics in Global Health
Source: Ann Glob Health. 2025 Mar 6;91(1):12. doi: 10.5334/aogh.4628 (PMC11887466; doi:10.5334/aogh.4628)
Supplement: Supplementary Table 1. — Glossary of Migration and References. [file agh-91-1-4628-s1.pdf]

Supplementary Materials

Table 1. – Glossary of Migration page 2
References page 4

Supplementary Table 1 – Glossary of Migration

**category**	**definition**	**consideration**
**migrant**	"An umbrella term, not defined under international law, reflecting the common lay understanding of a person who moves away from his or her place of usual residence, whether within a country or across an international border, temporarily or permanently, and for a variety of reasons. The term includes a number of well-defined legal categories of people"[1]	Many studies focused on the category of migrant. This highlights the overall peculiarity of these subjects and it somehow credits the challenging condition related to the act of living in a social environment other than the one of origin. This condition deprived the individual of many of the social and cultural aspects which underlie a person's existence. Nevertheless, in some cases authors fail to account that the challenging living conditions are linked by multifaceted factors, which burden the individual in a different way. One of the main problems of this approach is that simplifying the migrant could minimize the socio-economic aspects which contribute to its condition. In this way, authors sometimes convey that the conditions under study are more related to the migration status than to social factors.[5,6]
**economic migrant**	"any person who is moving or has moved across an international border or within a State, solely or primarily motivated by economic opportunities"[2]	When the migration plan is so bound to working and economical activity, work market changings play a pivotal role in the quality of life of the migrant. Thus, the access to welfare could change either according to local workfare and according to work stability.[7,8]
**migrant worker**	"a person who is to be engaged, is engaged or has been engaged in a remunerated activity in a State of which he or she is not a national."[2]	
**long-term migrant**	an individual who has moved to a country other than their usual residence for a period of at least 12 months, so that the country of destination can be considered the new country of usual residence.[3]	Interestingly, long-term migrant conditions present cross-sectional characteristics between non-migrants and migrants. On one hand their social integration spare them from some of the typical characteristics of migrant-related demarginalization, on the other hand they persist for some aspects as foreign people in a host society.[9]
**asylum seeker**	"an individual who is seeking international protection. In countries with individualized procedures, an asylum seeker is someone whose claim has not yet been finally decided on by the country in which he or she has	Refugee status or asylum seeker status present different aspects which could balance the access to accurate care. In many countries, these two categories represent a target for specific welfare laws. Nevertheless,

	submitted it. Not every asylum seeker will ultimately be recognized as a refugee, but every recognized refugee is initially an asylum seeker."[2]	the recent traumatic experience and the precarious life condition enhance the difficulties related with migration status, which affect the general quality of life[5, 10]
**refugee**	A person who, owing to a well-founded fear of persecution for reasons of race, religion, nationality, membership of a particular social group or political opinion, is outside the country of his nationality and is unable or, owing to such fear, is unwilling to avail himself of the protection of that country; or who, not having a nationality and being outside the country of his former habitual residence as a result of such events, is unable or, owing to such fear, is unwilling to return to it[2]	
**climate refugee/disaster refugee**	persons obliged to leave their habitual homes because of environment or climate change or natural disaster related reasons[2]	
**internal displaced people**	"Persons or groups of persons who have been forced or obliged to flee or to leave their homes or places of habitual residence, in particular as a result of or in order to avoid the effects of armed conflict, situations of generalized violence, violations of human rights or natural or human-made disasters, and who have not crossed an internationally recognized State border."[2]	The phenomenon of Internal displaced people is generally related to emergency and crisis conditions; both climate-related disaster and conflict concur to destabilize migrant life conditions. The quality of access to healthcare is generally affected and the response capacity of government, institutions, NGO or other stakeholders, is the factor which mostly could make a difference. This underline how self-determination is highly reduced in this category.[5,11,12]
**unaccompanied foreign minors**	foreign children "who have been separated from both parents and other relatives and are not being cared for by an adult who, by law or custom, is responsible for doing so"[2]	Unaccompanied minors are more vulnerable to inaccurate caring since their care is often istitutionalised or lack a stable referring figure.[5]
**second generation migrant**	progeny of migrant[4]	The inclusion of progeny of migrants in the migrant population is controversial and the attitude is heterogeneous among the different authors. Many aspects can be involved in the characterisation of this category. If a second generation migrant or a child migrant experienced the major part of personal development merge into the host society, many aspects of personal life are affected by the experience of migration, either direct for child migrant or indirect and mirrored by parents' one for second-generation migrant. Nonetheless, the migrant family more easily expose the individual to belonging to a low-income family.[13]
**child migrant**	individuals who migrate to another country in their early year of life and for this reason their personal development is associated with host country culture[2]	
**undocumented**	"a non-national who enters or stays in a	irregular status is related with precarious life

**migrant**	country without the appropriate documentation"[2]	conditions which affect the programming capacity and the possibility to perform routinary life activity.[14]

References

(1) International Organization for Migration. Who is a migrant? [Internet]. 2021 [cited 2024 Aug 12]. Available from: https://www.iom.int/who-migrant-0

(2) International Organization for Migration. Glossary on migration. *IML Series No. 34.* 2019.

(3) United Nations, Department of Economic and Social Affairs. Recommendations on Statistics of International Migration, Revision 1. New York: United Nations; 1998.

(4) United Nations, Department of Economic and Social Affairs, Population Division. International Migration Report 2020. New York: United Nations; 2020.

(5) Lebano A, Hamed S, Bradby H, et al. Migrants' and refugees' health status and healthcare in Europe: a scoping literature review. *BMC Public Health.* 2020;20(1):1039. doi:10.1186/s12889-020-08749-8. PMID: 32605605; PMCID: PMC7329528.

(6) van Rosse F, de Bruijne M, Suurmond J, Essink-Bot ML, Wagner C. Language barriers and patient safety risks in hospital care. A mixed methods study. *Int J Nurs Stud.* 2016;54:45-53. doi:10.1016/j.ijnurstu.2015.03.012. PMID: 25840899.

(7) Porru S, Baldo M. Occupational health and safety and migrant workers: has something changed in the last few years? *Int J Environ Res Public Health.* 2022;19(15):9535. doi:10.3390/ijerph19159535. PMID: 35954890; PMCID: PMC9367908.

(8) Hargreaves S, Rustage K, Nellums LB, et al. Occupational health outcomes among international migrant workers: a systematic review and meta-analysis. *Lancet Glob Health.* 2019;7(7):e872-e882. doi:10.1016/S2214-109X(19)30204-9. PMID: 31122905; PMCID: PMC6565984.

(9) De Haas H. Migration and development: a theoretical perspective. *Int Migr Rev.* 2010;44(1):227-264. doi:10.1111/j.1747-7379.2009.00804.x

(10) Bradby H, Humphris R, Newall D, Phillimore J. Public Health Aspects of Migrant Health: A Review of the Evidence on Health Status for Refugees and Asylum Seekers in the European Region. Copenhagen: WHO Regional Office for Europe; 2015. PMID: 27536765.

(11) Ehiri JE, Gunn JK, Center KE, Li Y, Rouhani M, Ezeanolue EE. Training and deployment of lay refugee/internally displaced persons to provide basic health services in camps: a systematic review. Glob Health Action. 2014 Oct 1;7:23902. doi: 10.3402/gha.v7.23902. PMID: 25280734; PMCID: PMC4185139.

(12) Orendain DJA, Djalante R. Ignored and invisible: internally displaced persons (IDPs) in the face of COVID-19 pandemic. Sustain Sci. 2021;16(1):337-340. doi:10.1007/s11625-020-00848-0

(13) Wallace M, Hiam L, Aldridge R. Elevated mortality among the second-generation (children of migrants) in Europe: what is going wrong? A review. Br Med Bull. 2023 Dec 11;148(1):5-21. doi: 10.1093/bmb/ldad027. PMID: 37933157; PMCID: PMC10724460.

(14) Onarheim KH, Melberg A, Meier BM, Miljeteig I. Towards universal health coverage: including undocumented migrants. BMJ Glob Health. 2018 Oct 8;3(5):e001031. doi: 10.1136/bmjgh-2018-001031. PMID: 30364297; PMCID: PMC6195153.
